# Mosquito microbiota cluster by host sampling location

**DOI:** 10.1186/s13071-018-3036-9

**Published:** 2018-08-14

**Authors:** Ephantus J. Muturi, Doris Lagos-Kutz, Christopher Dunlap, Jose L. Ramirez, Alejandro P. Rooney, Glen L. Hartman, Christopher J. Fields, Gloria Rendon, Chang-Hyun Kim

**Affiliations:** 1 Crop Bioprotection Research Unit, Agricultural Research Service, U.S. Department of Agriculture, 1815 N. University St., Peoria, IL 61604 USA; 2 National Soybean Research Center, Agricultural Research Service,U.S. Department of Agriculture, 1101 W. Peabody Dr., Urbana, IL 61801 USA; 3 High Performance Biological Computing (HPCBio), Roy J Carver Biotechnology Center, University of Illinois at Urbana-Champaign, 1206 West Gregory Dr., Urbana, IL 61801 USA; 4 Illinois Natural History Survey, University of Illinois at Urbana-Champaign, 1816 S. Oak St., Champaign, IL 61820 USA

**Keywords:** Mosquitoes, Microbiota, HiSeq, *16S* rRNA gene

## Abstract

**Background:**

Microbial communities that inhabit the mosquito body play an import role in host biology and may have potential for mosquito control. However, the forces that shape these microbial communities are poorly understood.

**Methods:**

To gain a better understanding of how host location influences the composition and diversity of mosquito microbiota, we performed a survey of microbial communities in mosquito samples collected from six USA states using HiSeq sequencing of the *16S* rRNA gene.

**Results:**

A total of 284 bacterial operational taxonomic units (OTUs) belonging to 14 phyla were detected in nine mosquito species, with Proteobacteria, Firmicutes and Actinobacteria accounting for 95% of total sequences. OTU richness varied markedly within and between mosquito species. The microbial composition and diversity was heavily influenced by the site of mosquito collection, suggesting that host location plays an important role in shaping the mosquito microbiota.

**Conclusions:**

Variation in microbial composition and diversity between mosquitoes from different locations may have important implications on vector competence and transmission dynamics of mosquito-borne pathogens. Future studies should investigate the environmental factors responsible for these variations and the role of key bacteria characterized in this study on mosquito biology and their potential application in symbiotic control of mosquito-borne diseases.

**Electronic supplementary material:**

The online version of this article (10.1186/s13071-018-3036-9) contains supplementary material, which is available to authorized users.

## Background

Animals harbor diverse microbial communities which have profound effects on host health. These microorganisms can range from disease-causing to commensal and mutually beneficial microbes. Insects in particular owe their evolutionary success and ecological diversification to their symbiotic associations with microorganisms [1]. Insects that feed on nutritionally deficient diets such as plant sap, woody materials and vertebrate blood, rely on obligate mutualistic microorganisms to degrade recalcitrant diet and to synthesize essential nutrients [2–4]. Microbial symbionts also play other roles in their hosts’ biology such as detoxification of compounds, protection against pathogens and natural enemies, heat tolerance, and mediating intra- and interspecific communication [4–9]. Aphids, tsetse flies and triatomine bugs are some of the well-studied insect systems with fascinating associations with microbial symbionts. Aphids feed exclusively on plant sap and harbor an endosymbiotic bacterium, *Buchnera aphidicola* that process essential amino acids that are limited in plant phloem [10]. Similarly, the microbial symbionts, *Wigglesworthia* spp. in tsetse flies and *Rhodococcus* spp. (e.g. *R. rhodnii*, *R. triatomae* and *R. corybacteriodes*) in triatomine bugs contribute to metabolism through synthesis of B complex vitamins that are deficient in blood diets [11–13].

Despite the significance of insect-associated microbiota in host physiology and evolution, our understanding of the factors that shape their composition and structure is limited. Accumulating evidence has demonstrated that insect-microbe interactions can be mediated by factors such as host phylogeny, diet, life stage, and infection by pathogens [14–16]. Host sampling location is also a key determinant of insect-associated microbiota as host exposure to different ecological conditions across study sites can alter within-host microbial composition and structure through direct effect on regional microbial pools [17] and indirectly, through effects on host fitness, abundance and nutritional quality [18].

Mosquitoes comprise around 3500 species worldwide and are among the most studied group of insects due to their role in transmission of diverse parasites and pathogens that cause diseases in humans, domestic animals and wildlife. Like other insects, mosquitoes host a diverse community of microorganisms that are known to influence host development, reproduction and susceptibility to pathogens [19–23]. These microorganisms may be used to control mosquito-borne parasites/pathogens such as malaria, dengue, chikungunya and Zika viruses, by reducing the mosquito lifespan [24], by blocking pathogen/parasite proliferation through natural competition mechanisms [25–29], or through expression of anti-pathogen molecules that are genetically introduced through paratransgenesis [30, 31]. The composition of mosquito microbiota can vary markedly between species and even among individuals of the same mosquito species [22, 32–34]. Although many studies have shown that factors such as host environment, stage of development, diet type, pathogenic infection and host sampling location can influence mosquito microbiota, these studies have mostly focused on a few mosquito species, primarily the vectors of malaria, dengue, Zika, chikungunya and yellow fever [15, 16, 35–37]. Thus, the microbiota of many mosquito species and the factors that shape their composition and diversity remain poorly understood.

Here, we surveyed the bacterial communities of adult mosquitoes originating from six states in the USA: Iowa, Louisiana, Michigan, Minnesota, Missouri and Wisconsin. The objective of the study was to develop a better understanding of how host location influences the composition and structure of mosquito microbiota. To achieve this, we used high-throughput next-generation sequencing to identify the bacterial microbes present in different mosquito species collected across the six states, and then compared the microbial community richness, diversity and composition within and between mosquito species to determine their association with host sampling location. The findings of this study improve our understanding of the factors that structure microbial communities in mosquitoes and provide an important first step in understanding mosquito-microbe interactions and identification of microbial taxa that can potentially be used for symbiotic control of mosquito-borne diseases.

## Methods

### Mosquito collection

This study took advantage of the suction trap network (STN) specifically established for sampling aphid populations. The suction trap network was first established in Illinois in 2001 and expanded in 2005 to most of the states in the Midwest of USA. Of the nine states under the STN, we were able to obtain mosquitoes from Iowa, Louisiana, Michigan, Minnesota, Missouri and Wisconsin (Table 1). Most of the traps were designed according to the protocol outlined by Allison & Pike [38], and built at the Illinois Natural History Survey (INHS) under the supervision of David Voegtlin. Each suction trap consisted of a ≈ 6 m vertical tube (30.5 cm diameter at the top and 38 cm at the bottom) with an electric fan drawing 10 m^3^ of air per minute. Although the traps were specifically designed to collect aphids, they also consistently collect mosquitoes, other dipterans, and other airborne abiotic and biotic agents that were sucked into the trap. Captured samples were drawn into a 250 ml Nalgene™ polypropylene jar (Thermo Fisher Scientific, Waltham, MA, USA) with a 70 mm opening containing 83 ml of a mix of 50% propylene glycol and 50% water. The propylene glycol works as an insect preservative and is not considered a hazardous liquid for shipping purposes [39]. The propylene glycol is also a proven preservative for microbial DNA from insect hosts and has been used in pitfall traps to collect insects for use in characterization of insect-associated microbiota [40, 41]. The suction traps were operated from May 20 through October 21, 2016 between 07:00 and 20:00 h. This timing enabled us to capture both day-biting and night-biting mosquito species. The jar was replaced weekly in each location. All the suction trap samples were shipped to the National Soybean Research Center, University of Illinois at Urban-Champaign where the aphids were picked out. The remaining samples were taken to the Medical Entomology Laboratory at the Illinois Natural History Survey, University of Illinois. Here the female mosquitoes were collected and preserved by location in 2 ml microcentrifuge tubes containing 500 μl of 95% ethanol and stored at -20 °C until DNA isolation.

**Table 1:** Number of mosquitoes of each species that were processed and their site of collection

Site	GPS coordinates and area description	Species	No. used for analysis	Total processed
Kanawha, Iowa: Northern Research Farm, Iowa State University	40.931, -93.80; Surrounding area rural, mostly corn and soybeans	*Ae. vexans*	16	16
		*Cx. pipiens*	5	5
		*Cx. tarsalis*	3	3
		Unclassified	0	5
Chase, Louisiana: Sweet Potato Research Station, Louisiana State University Agricultural Center	32.101, -91.703; Surrounding area rural, mostly crops including some corn and soybeans	*An. quadrimaculatus*	6	7
		*Cx. quinquefasciatus*	2	2
		*Mn. titillans*	1	1
		*Ps. confinnis*	6	7
		Unclassified	0	13
East Lansing, Michigan: South Campus Field Research Facilities, Michigan State University	42.691, -84.498; Surrounding area mixed with cityscape and some experimental plots of various crops	*Ae. vexans*	27	27
		*Cx. pipiens*	3	3
		Unclassified	0	0
Morris, Minnesota: West Central Research and Outreach Center	44.706, -95.869; Surrounding area rural mostly corn and soybeans	*Cx. pipiens*	21	24
		Unclassified	0	5
Columbia, Missouri: Campus, University of Missouri	38.907, -92.281; Surrounding area mixed with some city scape and rural mostly corn and soybeans	*Cx. pipiens*	15	20
		*Cx. tarsalis*	1	1
		Unclassified	0	9
Rhinelander, Wisconsin: Rhinelander Agriculture Research Station, University of Wisconsin	45.663, -89.269 Surrounding area forested with some fields of potato	*Cs. melanura*	9	10
		*Cx. salinarius*	1	2
		Unclassified	0	3
Total			116	163

### Sample processing and DNA extraction

A total of 163 mosquito samples were processed for microbial analysis (Table 1). Each individual female mosquito was rinsed once in 70% ethanol for 10 min and 5 times in sterile Dulbecco’s phosphate-buffered saline (DPBS) solution (Thermo Fisher Scientific) for 10 s and placed in a 1.5 ml microcentrifuge tube containing 100 μl sterile DPBS solution. A 3.2 mm stainless steel bead was added in the tube and the whole mosquito was macerated by Tissuelyser II (Qiagen, Valencia, CA, USA) at 28 beats/s for 4 min. The entire lysate was utilized for genomic DNA isolation using QIAamp DNA mini kit (Qiagen) following the manufacturer’s instructions. Isolated DNA was reconstituted in 100 μl of AE buffer and two aliquots of 50 μl each were prepared and stored at -20 °C until further processing. One 50-μl aliquot of the resulting DNA isolate was utilized for mosquito species identification because morphological identification was impossible due to the loss of morphological features during sample collection. The other 50-μl aliquot of the DNA isolate was used to build a microbiome library for Illumina HiSeq sequencing at the W.M. Keck Center for Comparative and Functional Genomics at the University of Illinois at Urbana-Champaign.

### Molecular identification of mosquitoes

PCR was performed to amplify the 5' cytochrome *c* oxidase subunit 1 (*cox*1) region of the mitochondrial DNA by using the primers of Kumar et al. [42]. The reaction was performed using Amplitaq Gold PCR mastermix (Thermo Fisher Scientific) with the following thermocycling parameters: step 1: 95 °C for 5 min; step 2: 5 cycles of 94 °C for 40 s, 45 °C for 1 min, and 72 °C for 1 min; step 3: 35 cycles of 94 °C for 40 s, 51 °C for 1 min, and 72 °C for 1 min; and step 4: 72 °C for 10 min. The amplification product was purified using Montage PCR Cleanup Filter Plates (Millipore, Billerica, MA, USA) and its size confirmed in a gel. Sequencing reactions for both forward and reverse strands were conducted using the ABI BigDye sequencing kit (Applied Biosystems, Foster City, CA, USA) following the manufacturer’s suggested protocol but at one-fourth the recommended volume. The reaction products were purified *via* ethanol precipitation and sequenced on an ABI3730 genetic analyzer (Applied Biosystems). The resulting DNA sequences were edited visually using Sequencher version 4.1.2 and identified by comparisons with the GenBank DNA sequence database at 97% sequence similarity [43]. Out of 163 mosquito samples that were tested, 133 were identified to species at 97% sequence similarity and were processed for analysis of microbiota as described below (Table 1).

### Preparation of *16S* rDNA gene library

All DNA samples were measured on a Qubit (Life Technologies, Grand Island, NY, USA) using High Sensitivity DNA Kit and diluted to 2 ng/μl prior to amplification. PCR amplification targeting the V3-V5 hypervariable region of bacterial *16S* rDNA was conducted using the methods and primer sequences described in Muturi et al. [33]. The final Fluidigm pools were transferred from the Functional Genomics laboratory to the DNA Services laboratory at the W. M. Keck Center at the University of Illinois at Urbana-Champaign where they were quantified using Qubit (Life Technologies) and qPCR on a BioRad CFX Connect Real-Time System (Bio-Rad Laboratories Inc., Hercules, CA, USA) and then pooled. The pool was mixed evenly based on the qPCR values, denatured and spiked with 16% indexed PhiX control library and loaded along with mosquito samples for other experiments onto 1 lane of the HiSeq V2 flowcell at a concentration of 9 pM for cluster formation and sequencing. The PhiX control library provides a balanced genome for calculation of matrix, phasing and prephasing, which are essential for accurate base-calling. The libraries were sequenced from both ends of the molecules to a total read length of 250 nt from each end. A negative control sample consisting of DNA extracted from molecular biology grade water was sequenced with the same metagenomic pipeline to allow empirical assessment of the contamination background.

### Operational taxonomic unit (OTU) picking and taxonomy assignment

The IM-TORNADO v.2.0.3.2 platform which is designed to process non-overlapping reads for analysis as a whole unit without sacrificing one of the reads in the pair, was used to process the de-multiplexed fastq-formatted files obtained from the sequencing facility [44]. The 5' PCR primer for forward (R1) and reverse (R2) reads were trimmed using Trimmomatic v.0.33 [45, 46] with the parameter HEADCROP:17 for R1 read and HEADCROP: 18 for R2 read. The quality filtering process, removal of chimeric sequences, and OTU picking process followed the same procedures described in our previous work [33, 47]. OTUs were assigned at 97% sequence similarity using the Ribosomal Database Project (RDP) version 10 as the reference set with a threshold of 70% bootstrap confidence [48].

### Statistical analyses

Statistical analyses were conducted using R 3.3.2 (https://cran.rproject.org/bin/windows/base-/old/3.2.3/) and PAST version 3.14 [49]. To reduce the problem of spurious OTUs, OTUs accounting for less than 0.005% of the total number of sequences were discarded before downstream analysis [50]. This procedure also removed the bacterial sequences that were detected in the negative control. Rarefaction and Shannon diversity curves were generated using the *phyloseq* package in R [51]. We also normalized the sequence number of each sample to 1213 to standardize the sampling effort. This step filtered out 17 samples and left 116 samples available for statistical analysis. Alpha diversity indices including Shannon diversity index (which accounts for both taxa abundance and evenness), number of observed OTUs (species richness), and Chao1 (taxa expected in sample based on extrapolation) were generated in QIIME v.1.9.1 [52] and their means and 95% confidence intervals were computed in R to test whether there were significant differences in taxa abundance and evenness between mosquito species and populations. Groups with non-overlapping confidence intervals were considered to be significantly different. Non-metric multidimensional scaling (NMDS) with Bray-Curtis similarity matrix values was computed using the *phyloseq* package to test whether microbial communities differed across mosquito species and study sites. Analysis of similarities (ANOSIM) computed using PAST was used to test whether microbial communities from the same mosquito species and study sites were more similar than those of different mosquito species and study sites. SIMPER analysis also performed in PAST was used to determine the bacterial taxa driving dissimilarity of microbial communities from different mosquito species and study sites.

## Results

A total of 4,906,732 raw sequences (both forward and reverse reads) were generated from the 163 mosquito samples that were sequenced. After removal of chimera reads and other non-bacterial sequences, a total of 1,292,622 sequences (mean ± SE = 7930.2 ± 488.2; minimum = 1, maximum = 38,582) were retained. A total of 133 mosquito samples out of the 163 analyzed were successfully assigned to one of the nine mosquito species: *Anopheles quadrimaculatus*, *Aedes vexans*, *Culex pipiens*, *Cx. quinquefasciatus*, *Cx. salinarius*, *Cx. tarsalis*, *Culiseta melanura*, *Mansonia titillans* and *Psorophora confinnis* (Table 1). Each mosquito species was collected at up to 3 sites (Table 1). After quality filtering of bacterial OTUs accounting for less than 0.005% of the total sequences, and rarefaction at 1213 sequences per sample, 116 mosquito samples were retained across the six study sites yielding a total of 284 bacterial OTUs. *Aedes vexans* (112–121 OTUs) had the largest number of OTUs while *Cx. quinquefasciatus* (14), *Cx. salinarius* (14), *Mn. titillans* (16) and *Cx. tarsalis* (16–33) had the lowest number of OTUs (Table 2). The low number of OTUs in the four mosquito species was likely due to the small sample size as only 1–3 individuals were tested for each species (Table 2). The majority of OTUs occurred in a few samples with 217 of the 284 bacterial OTUs occurring in 10 or fewer mosquito samples, 94 of which occurred in a single mosquito (Additional file 1: Table S1). The most prevalent OTUs were OTU 7 (*Propionibacterium*), OTU 4 (*Bacillus*), OTU 5 (*Pseudomonas*), OTU 12 (*Pseudomonas*), and OTU 9 (*Acinetobacter*) occurring in 60, 55, 53, 53 and 50 mosquito samples, respectively.

**Table 2:** Diversity and richness (mean and 95% confidence limits) of whole-body bacterial communities of nine mosquito species from six states in the USA

State	Species	*n*	No. of OTUs	Shannon index	Observed OTUs	Chao1
Iowa	*Ae. vexans*	16	112	2.56 (2.14–2.98)	19.31 (14.15–24.48)	20.67 (15.10–26.23)
Iowa	*Cx. pipiens*	5	61	3.13 (1.96–4.30)	25.00 (7.29–42.71)	27.84 (9.07–46.62)
Iowa	*Cx. tarsalis*	3	33	2.63 (1.61–3.65)	13.67 (-8.60–35.93)	14.78 (-12.26–41.82)
Louisiana	*An. quadrimaculatus*	6	54	2.29 (1.85–2.73)	13.00 (6.60–19.40)	13.50 (6.97–20.03)
Louisiana	*Cx. quinquefasciatus*	2	14	1.86 (-0.14–3.86)	10.00 (-15.41–35.41)	10.50 (-8.56–29.56)
Louisiana	*Mn. titillans*	1	16	2.17	16.00	17.00
Louisiana	*Ps. confinnis*	6	68	2.55 (1.65–3.45)	14.67 (7.02–22.31)	16.42 (4.62–28.22)
Michigan	*Ae. vexans*	27	121	3.32 (3.17–3.46)	36.56 (34.73–38.39)	46.54 (42.29–50.79)
Michigan	*Cx. pipiens*	3	62	3.42 (3.13–3.71)	36.00 (27.39–44.61)	39.95 (27.70–52.20)
Minnesota	*Cx. pipiens*	21	85	2.14 (1.77–2.51)	9.00 (6.86–11.14)	9.43 (6.85–12.01)
Missouri	*Cx. pipiens*	15	86	2.74 (2.21–3.26)	15.27 (11.20–19.33)	16.59 (11.97–21.21)
Missouri	*Cx. tarsalis*	1	16	3.27	16.00	16.00
Wisconsin	*Cs. melanura*	9	39	2.03 (1.60–2.46)	8.56 (5.63–11.49)	8.59 (5.64–11.54)
Wisconsin	*Cx. salinarius*	1	14	2.68	14.00	15.50

**Fig. 1:**
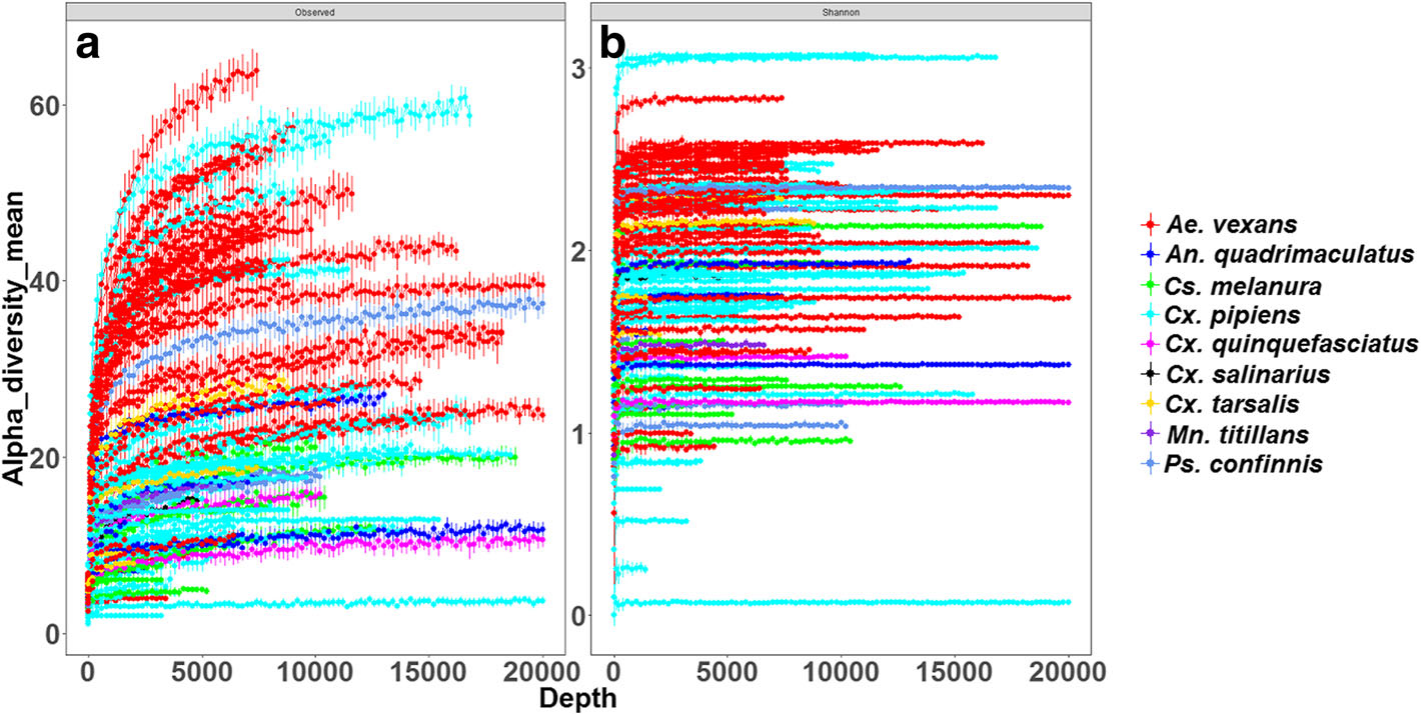
Rarefaction analysis of observed richness (**a**) and Shannon index (**b**) within individual mosquitoes

Rarefaction analysis showed that microbial richness and diversity vary among individual mosquitoes and that the libraries were sampled at different depths (Fig. 1). Rarefaction curves for some individual mosquito samples did not plateau indicating the potential for unrecovered rare bacterial taxa. To determine whether bacterial diversity and richness varied significantly between mosquito species and study sites, we computed the alpha diversity indices along with 95% confidence intervals (Table 2). Results revealed that bacterial diversity was significantly higher among *Cx. pipiens* and *Ae. vexans* from Michigan compared to *Cx. pipiens* from Minnesota, *Ae. vexans* from Iowa, *An. quadrimaculatus* from Louisiana and *Cs. melanura* from Wisconsin. Bacterial richness was also significantly higher among *Cx. pipiens* and *Ae. vexans* from Michigan compared to *Ae. vexans* from Iowa, *Cx. pipiens* from Missouri and Minnesota, *Cs. melanura* from Wisconsin, and *An. quadrimaculatus* and *Ps. confinnis* from Louisiana. In addition, *Cx. pipiens* from Missouri had significantly higher bacterial richness compared to *Cx. pipiens* from Minnesota and *Cs. melanura* from Wisconsin.

**Fig. 2:**
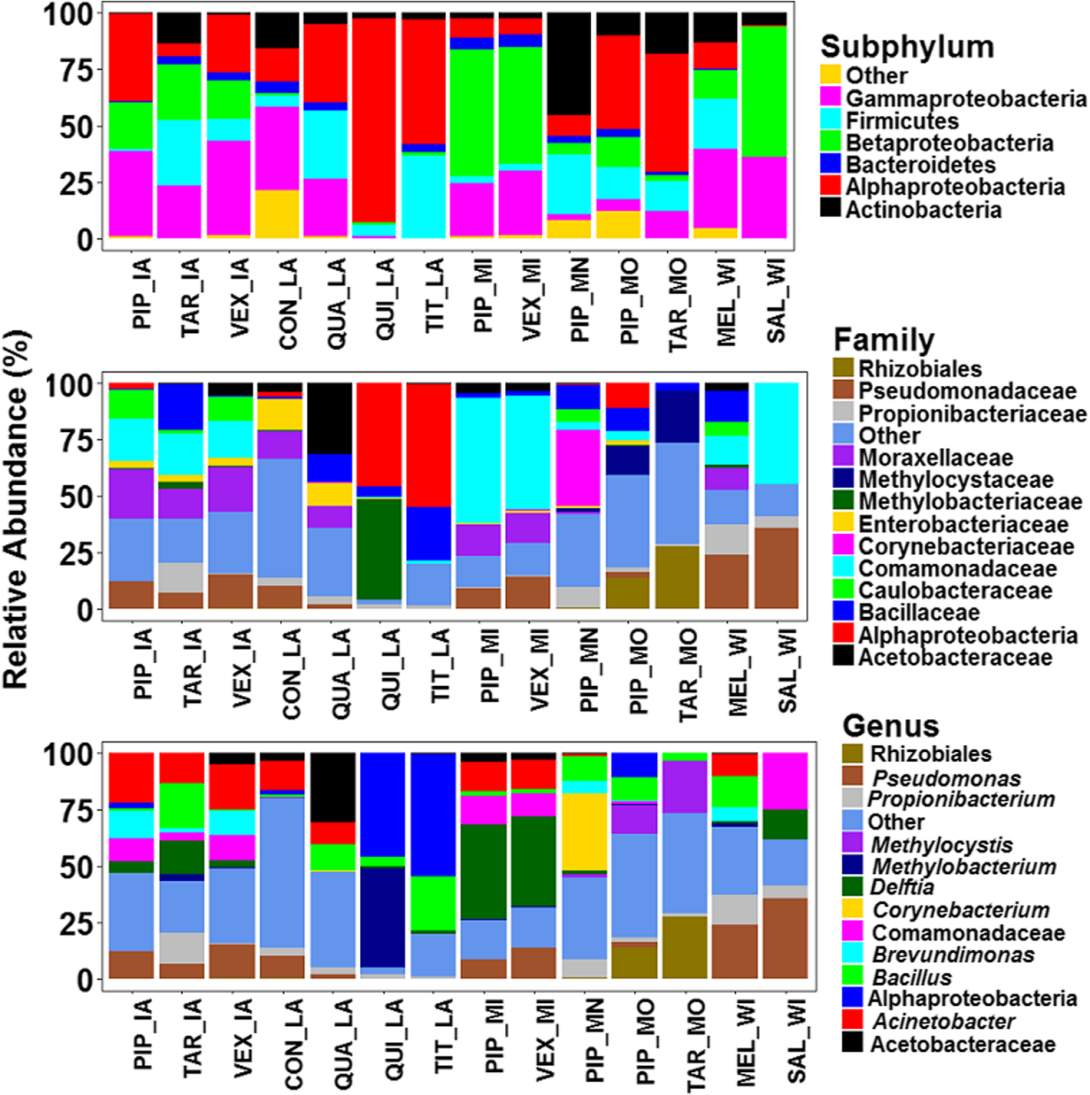
Relative abundances of bacterial communities detected in adult females from six USA states. Bacterial phyla with abundance of less than 1% and bacterial families and genera with abundance of less than 2% were pooled together as “Other”. *Abbreviations*: PIP, *Culex pipiens*; QUI, *Cx. quinquefasciatus*; TAR, *Cx. tarsalis*; SAL, *Cx. salinarius*; QUA, *Anopheles quadrimaculatus*; VEX, *Aedes vexans*; MEL, *Culiseta melanura*; CON, *Psorophora confinnis*; and TIT, *Mansonia titillans*; MI, Michigan; IA, Iowa; MO, Missouri; MN, Minnesota; WI, Wisconsin; LA, Louisiana

A total of 14 bacterial phyla were identified in this study (Additional file 2: Table S2). The most dominant phyla were Proteobacteria (70.7%), Firmicutes (14.1%), Actinobacteria (10.2%) and Bacteroidetes (2.8%) which collectively accounted for 97.8% of the total sequences. Among the Proteobacteria, the most dominant taxon was Alphaproteobacteria (28.2%) followed by Gammaproteobacteria (22.0%) and Betaproteobacteria (18.9%). The relative abundance of these taxa varied markedly between mosquito samples (Fig. 2). Alphaproteobacteria was the most abundant taxon in *Cx. pipiens* and *Cx. tarsalis* from Missouri, *Cx. pipiens* from Iowa, and *Cx. quinquefasciatus*, *An. quadrimaculatus* and *Mn. titillans* from Louisiana. Betaproteobacteria was the most abundant taxon in *Cx. pipiens* and *Ae. vexans* from Michigan, and *Cx. salinarius* from Wisconsin. Gammaproteobacteria was either the first or the second most abundant taxon in *Cx. pipiens* and *Ae. vexans* from Michigan and Iowa, *Cx. salinarius* and *Cs. melanura* from Wisconsin and *Ps. confinnis* from Louisiana. Actinobacteria was the most abundant taxon in *Cx. pipiens* from Minnesota and Firmicutes was mostly found in *Cx. pipiens* from Minnesota, *Cx. tarsalis* from Iowa, *Cs. melanura* from Wisconsin, and *Mn. titillans* and *An. quadrimaculatus* from Louisiana. This pattern continued at the family and genus level, where on average, mosquitoes from the same study site tended to have more similar microbiota compared to those from different sites (Fig. 2). The family Methylocystaceae and an unclassified family from the order Rhizobiales were mostly detected in mosquitoes from Missouri while Comamonadaceae was mostly detected in mosquitoes from Wisconsin and Michigan, and to a lesser extent in mosquitoes from Iowa. Corynebacteriaceae was mostly found in *Cx. pipiens* from Minnesota, Methylobacteriaceae in *Cx. quinquefasciatus* from Louisiana and Acetobacteriaceae in *An. quadrimaculatus* from Louisiana. An unclassified family of Alphaproteobacteria was also common in *Cx. quinquefasciatus* and *Mn. titillans* from Louisiana. Pseudomonadaceae was more abundant in mosquito samples from Wisconsin while Enterobacteriaceae were more abundant in *An. quadrimaculatus* and *Ps. confinnis* from Louisiana.

At the genus level, *Methylocystis* and an unclassified genus from the order Rhizobiales were mostly detected in mosquitoes from Missouri, *Delftia* in mosquitoes from Michigan, *Pseudomonas* in mosquitoes from Wisconsin, *Corynebacteria* in *Cx. pipiens* from Minnesota, while *Bacillus* was mostly detected in *Mn. titillans* from Louisiana and *Cx. tarsalis* from Iowa (Fig. 2). In addition, *Acinetobacter* was mostly common in mosquitoes from Michigan and Iowa as well as in *Cs. melanura* from Wisconsin and *Ps. confinnis* and *An. quadrimaculatus* from Louisiana.

**Fig. 3:**
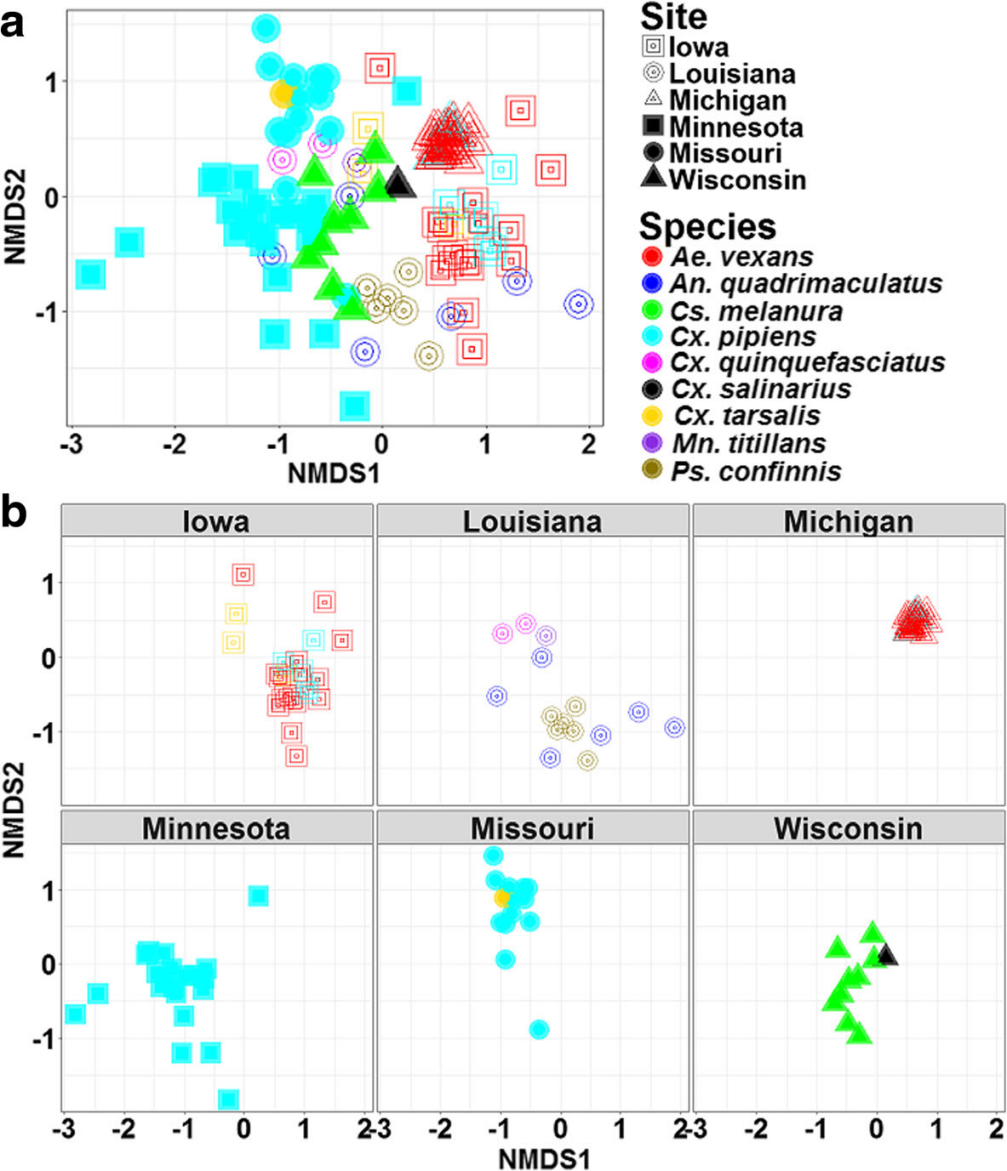
Non-metric multidimesional scaling (NMDS) ordination of Bray-Curtis distances between microbial communities of nine mosquito species from six states. The states are presented together (**a**) and separately (**b**)

Nonmetric multidimensional scaling (NMDS) analysis of the OTUs revealed considerable variation in microbial communities according to species and site of collection (Fig. 3). Samples from Iowa and Michigan clustered on the positive side of MDS 1 while samples from Missouri, Wisconsin, and Minnesota clustered on the negative side of MDS 1. This difference was significant with a non-parametric permutation analysis of similarity (ANOSIM) test (*R* = 0.699, *P* = 0.0001). Pairwise ANOSIM revealed 21 significant pairwise comparisons, which reduced to 12 significant pairwise comparisons after Bonferroni correction for multiple comparisons (Table 3, Additional file 3: Table S3). The community structure of microbiota of *Ae. vexans* from Michigan was substantially different from that of *Cs. melanura* from Wisconsin, *An. quadriamaculatus* and *Ps. confinnis* from Louisiana and *Cx. pipiens* from Iowa (*R* statistics range 0.81–0.87). Varying degree of overlap but generally different community structure was observed between microbiota of *Ae. vexans* from Michigan and *Ae. vexans* from Iowa and *Cx. pipiens* from both Missouri and Minnesota. A similar pattern of microbiota was observed between *Cs. melanura* from Wisconsin and *Cx. pipiens* from Missouri and between *Ae. vexans* from Iowa and *Cs. melanura* from Wisconsin and *Cx. pipiens* from both Missouri and Minnesota (*R* statistics range 0.27–0.74). There was little separation between the microbiota of *Cx. pipiens* from Missouri and Minnesota (*R* statistic = 0.21).

**Table 3:** Pairwise ANOSIM comparisons of sample groups. Of the 21 pairwise comparisons that were significantly different from each other, 12 remained significant after adjusting for multiple comparisons; only these are shown

Pairwise comparison	Global *R*	*P*-value
VEX_MI *vs* VEX_IA	0.660	0.0001
VEX_MI *vs* QUA_LA	0.867	0.0001
VEX_MI *vs* MEL_WI	0.821	0.0001
VEX_MI *vs* PIP_MO	0.735	0.0001
VEX_MI *vs* PIP_IA	0.813	0.0001
VEX_MI *vs* PIP_MN	0.594	0.0001
VEX_MI *vs* CON_LA	0.855	0.0001
VEX_IA *vs* MEL_WI	0.342	0.0001
VEX_IA *vs* PIP_MO	0.285	0.0001
VEX_IA *vs* PIP_MN	0.269	0.0001
MEL_WI *vs* PIP_MO	0.366	0.0005
PIP_MO *vs* PIP_MN	0.208	0.0006

*Abbreviations*: *PIP* *Culex pipiens*, *QUA* *Anopheles quadrimaculatus*, *VEX* *Aedes vexans*, *MEL* *Culiseta melanura*, *CON* *Psorophora confinnis*, *MI* Michigan, *IA* Iowa, *MO* Missouri, *MN* Minnesota, *WI* Wisconsin, *LA* Louisiana

Total dissimilarity between pairs of mosquito species from different collection sites and the relative contribution of each bacterial OTU to the observed dissimilarity was determined by SIMPER analysis. The results revealed an overall average dissimilarity of 94.2% among all mosquito-site pairs. A range of OTUs contributed to differences between samples (Additional file 4: Table S4). The OTUs that contributed most to the observed differences were OTU 2 (*Delftia*, 9.0%), OTU 4 (*Bacillus*, 4.4%), OTU 10 (*Corynebacterium*, 4.0%), OTU 3 (*Acinetobacter*, 3.6%), OTU 13 (*Brevundimonas*, 3.3%) and OTU 7 (*Propionibacterium*, 3.3%). OTU 2 was more abundant in *Cx. pipiens* and *Ae. vexans* samples from Michigan. OTU 4 was more abundant in *Cx. tarsalis* from Iowa, *Mn. titillans* and *An. quadrimaculatus* from Louisiana, *Cx. pipiens* from Missouri and Minnesota and *Cs. melanura* from Wisconsin. OTU 10 was highly abundant in *Cx. pipiens* from Minnesota. OTU 3 was highly abundant in *An. quadrimaculatus* from Louisiana and *Ae. vexans*, *Cx. pipiens* and *Cx. tarsalis* from Iowa. OTU 13 was highly abundant in *Ae. vexans* and *Cx. pipiens* samples from Iowa and OTU 7 was abundant in *Cs. melanura* from Wisconsin and *Cx. tarsalis* from Iowa.

## Discussion

Although it is well known that blood-feeding arthropods such as mosquitoes exhibit a variety of microbial associations, many questions remain about what factors shape host-associated microbial composition and diversity. The objective of this study was to determine the role of host sampling location in shaping the pattern of mosquito-associated microbiota. Unlike our previous studies that focused on the microbiota of mosquito species collected within a small geographical area in Champaign County Illinois [32, 36, 53], mosquito samples for this study were sampled from six states in continental USA. Each of the nine mosquito species was collected in one to three study sites, making it impossible to examine how the microbiota of each species compared across the six study sites. However, even with this limitation, we believe our findings add to the limited knowledge on the microbiota of different mosquito species, and provides some important insights into how host location influences the composition and diversity of mosquito microbiota. Overall, we determined that the whole-body microbiota of mosquitoes was strongly influenced by the site in which the mosquitoes were collected and in some cases by the mosquito species. This microbiota was dominated by Proteobacteria (70.7%) and Firmicutes (14.1%) which is consistent with previous studies [32, 33, 35, 54]. Actinobacteria was also dominant in some mosquito samples which might occur due to variations in physiological conditions of individual mosquitoes and/or the environmental conditions they experience.

The strong effect of host sampling location on microbial composition and diversity in mosquitoes might be viewed as an approximation for the sum of environmental effects such as local weather patterns, availability of certain food sources (e.g. blood-meal and nectar sources), and other biotic and abiotic factors. Previous studies have shown that the environment of the sampling site is a key determinant of the bacterial profiles that colonize the mosquitoes [35, 37, 54–56]. These bacterial species could be acquired from the larval environment [35], vertebrate blood-meal hosts, and vegetation that serve as food sources and resting sites for adult mosquitoes [54]. Indeed, the most abundant OTUs in this study are commonly found in mosquitoes [32, 33, 57–59] and occur in at least one of these habitats. *Delftia*, *Methylobacterium*, *Acinetobacter*, and members of Acetobacteraceae and Comamonadaceae are frequently associated with plants [60, 61]. *Corynebacterium* is associated with skin of humans and animals, and *Bacillus*, *Pseudomonas*, *Acinetobacter* and *Methylocystis* are generalists that occur in both aquatic and terrestrial environments in association with both plants and animals [60–65]. *Acinetobacter* is particularly common in nectar where its relative abundance ranges between 49–90% in some plants [60]. Both male and female mosquitoes feed on nectar and would be expected to acquire this bacterium while nectar feeding. *Pseudomonas*, a common bacterial genus in mosquito larval habitats has been shown to colonize the Malpighian tubules of the mosquito larvae facilitating its transstadial passage from larvae to adult [66, 67]. Thus, similar to what has been reported for *Drosophila* species, different environments may harbor different types of bacteria and mosquitoes from different environmental niches may acquire different types of bacteria [68]. Alternatively, bodies of different mosquito species exposed to the same environmental conditions may selectively favor the growth and survival of the same bacterial taxa [68]. This is especially likely to occur if some of these microbial taxa confer some adaptation to local environmental conditions. We did not investigate the environmental factors that our mosquito samples may have been exposed to, nor the possible roles of detected microbes on host adaptation to local environmental conditions. Future studies incorporating these aspects and focusing on both immature and adult stages of mosquitoes across geographic regions could be more revealing.

It is also noteworthy that similarities in microbial communities among mosquito species from the same study sites could result from cross-contamination during sample collection and processing. Our samples were collected and held for one week in 50% propylene glycol, an insect preservative [39], prior to transfer to 95% ethanol, which may have presented an opportunity for cross-contamination. This method, combined with surface sterilization of samples in 70% ethanol and sterile water, has been used in studies on ant-microbe interactions without compromising the interpretation of results [41]. In fact, propylene glycol has been shown to be a suitable alternative for preserving insect samples for future use in DNA-based research on the host or host-associated bacteria when ethanol is not readily available [40, 69, 70]. Although we cannot rule out the potential for contamination despite having performed surface-sterilization to individual mosquitoes to remove surface bacteria, we are confident our results are a true reflection of the strong impact of sampling location on mosquito microbiota. Further evidence that contamination was not an issue is provided by the marked variation in bacterial OTU richness between mosquito species collected from the same study sites. In the Iowa study site for example, 112 bacterial OTUs were detected in *Ae. vexans* compared to 61 OTUs in *Cx. pipiens* and 33 OTUs in *Cx. tarsalis*. This trend was replicated in the other study sites. Thus, it is clear from these results that variation in microbiota between mosquito species may occur in some sampling locations but not in others, which is consistent with previous findings [32–34, 55].

The role of the major bacterial genera identified in this study on mosquito biology remains poorly understood but a few observations have been made. The genus *Bacillus* is thought to be essential for mosquito reproduction [71] and *Bacillus* spp. are involved in the digestion of polysaccharides and aromatic compounds such as chitin and lignocellulose in termites [72]. *Pseudomonas aeruginosa* was shown to improve the growth of *Cx. quinquefasciatus* larvae in a phosphorus-rich medium but inhibited the growth of *Cx. tarsalis*, suggesting that members of this bacterial genera may play an important role on mosquito adaptation to hypereutrophic aquatic habitats [73]. *Corynebacterium* spp. are key components of vertebrate host skin microbiota and are known to produce volatile compounds that attract host-seeking mosquitoes [62]. *Acinetobacter* spp. is likely involved in nectar assimilation and blood meal digestion as isolates of this genus from mosquitoes are able to metabolize components of both blood and plant sap [74].

Our results are consistent with previous findings that mosquitoes have low bacterial richness [33, 34, 55]. Although the total number of bacterial OTUs identified in different mosquito species ranged between 14–121, individual mosquitoes harbored a much lower number of bacterial OTUs averaging 9–37 OTUs, and many OTUs occurred in a few individuals. Most of the nine mosquito species identified in this study occurred in only one of the six study sites, and further studies examining multiple populations of each mosquito species are likely to discover some additional bacterial taxa. Given the well-documented role of microbial symbionts in mosquito nutrition, survival and susceptibility to pathogens [19–23, 75], the observed variation in microbial composition and diversity between mosquito populations is likely a key determinant of population variation in the ability of mosquitoes to transmit pathogens. Further studies are needed to determine the role of various bacterial taxa identified in this study on mosquito biology with the ultimate goal of identifying those that could be harnessed for mosquito-borne disease control.

The endosymbiont *Wolbachia* is commonly reported in mosquitoes [32, 33, 54, 76] and is known to induce a variety of reproductive phenotypes in their hosts such as cytoplasmic incompatibility, male-killing, parthenogenesis and feminization, to facilitate their spread into the host population [77–79]. Interestingly, *Wolbachia* was not detected in any of the mosquito samples in our study despite being one of the dominant bacterial taxa identified in *Cx. pipiens* in our recent studies in Champaign County, Illinois [32, 33]. It is unclear why *Wolbachia* was conspicuously absent in our samples, but we can offer some possible explanations. First, though unlikely, it is possible that mosquito populations from the six study sites were *Wolbachia*-free as indicated by our results. Alternatively, the mosquito samples for this study were collected using suction traps compared to CDC light traps and BG-Sentinel traps that are commonly used in other studies [32, 33, 76]. It is possible that *Wolbachia* was present in mosquito populations from these study sites but suction traps selectively attracted and captured *Wolbachia*-free mosquitoes. There is some evidence from *Drosophila* that *Wolbachia* can influence behaviors related to olfaction [80, 81]. Future studies examining the prevalence of different bacterial taxa including *Wolbachia* in mosquito samples collected using suction traps (with propylene glycol) and other sampling methods may shed light on the potential impact of propylene glycol on these microbes. Finally, although propylene glycol is known to be a suitable preservative for microbial DNA from insect hosts [40], it is possible it may have differential effect on the stability of DNA from intracellular (e.g. *Wolbachia*) *versus* extracellular bacteria. Further studies are needed to determine which of these factors are responsible for the absence of *Wolbachia* in our mosquito samples.

## Conclusions

In summary, this study provides a unique perspective on the microbial composition and diversity in nine mosquito species and how they are influenced by host location. This knowledge could be of value to vector biologists with interest in exploiting microbial functions to control mosquito-borne diseases. In addition to expanding the number of mosquito species whose microbiota has been characterized, this study opens an opportunity for additional studies to identify the key environmental factors responsible for site-specific variations in mosquito microbiota. Among the factors that should be included in future studies include host genetics, climatic factors, presence of alternative vertebrate hosts, composition and structure of vegetation, among other biotic and abiotic variables. Future studies should also examine the contribution of various bacterial taxa identified in this study on vector susceptibility to a variety of pathogens and the potential to harness these bacterial taxa for symbiotic control of mosquito-borne diseases. These studies should also interrogate how site-specific variation in mosquito microbiota may impact the success of microbe-based mosquito-borne disease control interventions.

## Additional files

Additional file 1: **Table S1.** Frequency of different bacterial OTUs in mosquito samples. (XLSX 11 kb)

Additional file 2: **Table S2.** Number of OTUs from each phylum that were identified from nine mosquito species from six USA states. (DOCX 13 kb)

Additional file 3: **Table S3.** Pairwise ANOSIM comparisons of sample groups. Only 12 pairwise comparisons (in bold) remained significant after adjusting for multiple comparisons. *Abbreviations*: PIP, *Culex pipiens*; QUA, *Anopheles quadrimaculatus*; VEX, *Aedes vexans*; MEL, *Culiseta melanura*; CON, *Psorophora confinnis*; MI, Michigan; IA, Iowa; MO, Missouri; MN, Minnesota; WI, Wisconsin; LA, Louisiana. Values on the upper half are global *R*-values while those on the lower half are the associated *P*-values. (DOCX 15 kb)

Additional file 4: **Table S4.** SIMPER analysis results showing differential abundance of bacterial OTUs among mosquito species from different study sites. (CSV 40 kb)

## Abbreviations

ABI: Applied Biosystems
*cox*1: cytochrome *c* oxidase subunit 1
DNA: Deoxyribonucleic acid
DPBS: Dulbecco’s phosphate-buffered saline
INHS: Illinois Natural History Survey
NMDS: Non-metric multidimensional scaling
OTUs: Operational taxonomic units
PCR: Polymerase chain reaction
STN: Suction trap network (STN)

## References

[1] Moran NA, Degnan PH (2006). Functional genomics of *Buchnera* and the ecology of aphid hosts. Mol Ecol..

[2] Brune A (2014). Symbiotic digestion of lignocellulose in termite guts. Nat Rev Microbiol..

[3] Sabree ZL, Kambhampati S, Moran NA (2009). Nitrogen recycling and nutritional provisioning by Blattabacterium, the cockroach endosymbiont. Proc Natl Acad Sci USA..

[4] Engel P, Moran NA (2013). The gut microbiota of insects - diversity in structure and function. FEMS Microbiol Rev..

[5] Dillon RJ, Vennard CT, Buckling A, Charnley AK (2005). Diversity of locust gut bacteria protects against pathogen invasion. Ecol Lett..

[6] Kikuchi Y, Hayatsu M, Hosokawa T, Nagayama A, Tago K, Fukatsu T (2012). Symbiont-mediated insecticide resistance. Proc Natl Acad Sci USA..

[7] Ceja-Navarro JA, Vega FE, Karaoz U, Hao Z, Jenkins S, Lim HC (2015). Gut microbiota mediate caffeine detoxification in the primary insect pest of coffee. Nat Commun..

[8] Wada-Katsumata A, Zurek L, Nalyanya G, Roelofs WL, Zhang A, Schal C (2015). Gut bacteria mediate aggregation in the German cockroach. Proc Natl Acad Sci USA..

[9] Tsuchida T, Koga R, Horikawa M, Tsunoda T, Maoka T, Matsumoto S (2010). Symbiotic bacterium modifies aphid body color. Science..

[10] Baumann P, Baumann L, Lai CY, Rouhbakhsh D, Moran NA, Clark MA (1995). Genetics, physiology, and evolutionary relationships of the genus *Buchnera*: intracellular symbionts of aphids. Annu Rev Microbiol..

[11] Moran NA, Plague GR, Sandstrom JP, Wilcox JL (2003). A genomic perspective on nutrient provisioning by bacterial symbionts of insects. Proc Natl Acad Sci USA..

[12] Pais R, Lohs C, Wu Y, Wang J, Aksoy S (2008). The obligate mutualist *Wigglesworthia glossinidia* influences reproduction, digestion, and immunity processes of its host, the tsetse fly. Appl Environ Microbiol..

[13] Beard CB, Cordon-Rosales C, Durvasula RV (2002). Bacterial symbionts of the triatominae and their potential use in control of Chagas disease transmission. Annu Rev Entomol..

[14] Yun JH, Roh SW, Whon TW, Jung MJ, Kim MS, Park DS (2014). Insect gut bacterial diversity determined by environmental habitat, diet, developmental stage, and phylogeny of host. Appl Environ Microbiol..

[15] Zouache K, Michelland RJ, Failloux AB, Grundmann GL, Mavingui P (2012). Chikungunya virus impacts the diversity of symbiotic bacteria in mosquito vector. Mol Ecol..

[16] Wang Y, Gilbreath TM, Kukutla P, Yan G, Xu J (2011). Dynamic gut microbiome across life history of the malaria mosquito *Anopheles gambiae* in Kenya. PLoS One..

[17] Cumming GS, Guegan JF (2006). Food webs and disease: is pathogen diversity limited by vector diversity?. EcoHealth..

[18] Keesing F, Holt RD, Ostfeld RS (2006). Effects of species diversity on disease risk. Ecol Lett..

[19] Cirimotich CM, Ramirez JL, Dimopoulos G (2011). Native microbiota shape insect vector competence for human pathogens. Cell Host Microbe..

[20] Ramirez JL, Short SM, Bahia AC, Saraiva RG, Dong Y, Kang S (2014). *Chromobacterium* Csp_P reduces malaria and dengue infection in vector mosquitoes and has entomopathogenic and in vitro anti-pathogen activities. PLoS Pathog..

[21] Coon KL, Brown MR, Strand MR (2016). Gut bacteria differentially affect egg production in the anautogenous mosquito *Aedes aegypti* and facultatively autogenous mosquito *Aedes atropalpus* (Diptera: Culicidae). Parasit Vectors..

[22] Coon KL, Vogel KJ, Brown MR, Strand MR (2014). Mosquitoes rely on their gut microbiota for development. Mol Ecol..

[23] Gaio Ade O, Gusmao DS, Santos AV, Berbert-Molina MA, Pimenta PF, Lemos FJ (2011). Contribution of midgut bacteria to blood digestion and egg production in *Aedes aegypti* (Diptera: Culicidae) (L.). Parasit Vectors..

[24] McMeniman CJ, Lane RV, Cass BN, Fong AWC, Sidhu M, Wang YF, O’Neill SL. Stable introduction of a life-shortening *Wolbachia* infection into the mosquito *Aedes aegypti*. Science. 2009;323:141–4. 10.1126/science.1165326 19119237

[25] Aliota MT, Peinado SA, Velez ID, Osorio JE (2016). The wMel strain of *Wolbachia* reduces transmission of Zika virus by *Aedes aegypti*. Sci Rep..

[26] Aliota MT, Walker EC, Uribe Yepes A, Velez ID, Christensen BM, Osorio JE (2016). The wMel strain of *Wolbachia* reduces transmission of chikungunya virus in *Aedes aegypti*. PLoS Negl Trop Dis..

[27] Moreira LA, Iturbe-Ormaetxe I, Jeffery JA, Lu GJ, Pyke AT, Hedges LM (2009). A *Wolbachia* symbiont in *Aedes aegypti* limits infection with dengue, chikungunya, and *Plasmodium*. Cell..

[28] Walker T, Johnson PH, Moreira LA, Iturbe-Ormaetxe I, Frentiu FD, McMeniman CJ (2011). The wMel *Wolbachia* strain blocks dengue and invades caged *Aedes aegypti* populations. Nature..

[29] Gonzalez-Ceron L, Santillan F, Rodriguez MH, Mendez D, Hernandez-Avila JE (2003). Bacteria in midguts of field-collected *Anopheles albimanus* block *Plasmodium vivax* sporogonic development. J Med Entomol..

[30] Riehle MA, Moreira CK, Lampe D, Lauzon C, Jacobs-Lorena M. Using bacteria to express and display anti-*Plasmodium* molecules in the mosquito midgut. Int J Parasitol. 2007;37:595–603. 10.1016/j.ijpara.2006.12.002 17224154

[31] Wang S, Ghosh AK, Bongio N, Stebbings KA, Lampe DJ, Jacobs-Lorena M (2012). Fighting malaria with engineered symbiotic bacteria from vector mosquitoes. Proc Natl Acad Sci USA..

[32] Muturi EJ, Kim CH, Bara J, Bach EM, Siddappaji MH (2016). *Culex pipiens* and *Culex restuans* mosquitoes harbor distinct microbiota dominated by few bacterial taxa. Parasit Vectors..

[33] Muturi EJ, Ramirez JL, Rooney AP, Kim CH (2017). Comparative analysis of gut microbiota of mosquito communities in central Illinois. PLoS Negl Trop Dis..

[34] Osei-Poku J, Mbogo CM, Palmer WJ, Jiggins FM (2012). Deep sequencing reveals extensive variation in the gut microbiota of wild mosquitoes from Kenya. Mol Ecol..

[35] Boissiere A, Tchioffo MT, Bachar D, Abate L, Marie A, Nsango SE (2012). Midgut microbiota of the malaria mosquito vector *Anopheles gambiae* and interactions with *Plasmodium falciparum* infection. PLoS Pathog..

[36] Muturi EJ, Bara JJ, Rooney AP, Hansen AK (2016). Midgut fungal and bacterial microbiota of *Aedes triseriatus* and *Aedes japonicus* shift in response to La Crosse virus infection. Mol Ecol..

[37] Buck M, Nilsson LK, Brunius C, Dabire RK, Hopkins R, Terenius O (2016). Bacterial associations reveal spatial population dynamics in *Anopheles gambiae* mosquitoes. Sci Rep..

[38] Allison D, Pike KS (1988). An inexpensive suction trap and its use in an aphid monitoring network. J Agric Entomol..

[39] Thomas DB (2008). Nontoxic antifreeze for insect traps. Entomol News..

[40] Moreau CS, Wray BD, Czekanski-Moir JE, Rubin BER (2013). DNA preservation: a test of commonly used preservatives for insects. Invertebr Syst..

[41] Hu Y, Holway DA, Lukasik P, Chau L, Kay AD, LeBrun EG (2017). By their own devices: invasive Argentine ants have shifted diet without clear aid from symbiotic microbes. Mol Ecol..

[42] Kumar NP, Rajavel AR, Natarajan R, Jambulingam P (2007). DNA barcodes can distinguish species of Indian mosquitoes (Diptera: Culicidae). J Med Entomol..

[43] GenBank DNA sequence database. National Center for Biotechnology Information. https://www.ncbi.nlm.nih.gov/. Accessed 2 Oct 2017.

[44] Jeraldo P, Kalari K, Chen X, Bhavsar J, Mangalam A, White B (2014). IM-TORNADO: a tool for comparison of 16S reads from paired-end libraries. PLoS One..

[45] Lohse M, Bolger AM, Nagel A, Fernie AR, Lunn JE, Stitt M, Usadel B (2012). RobiNA: a user-friendly, integrated software solution for RNA-Seq-based transcriptomics. Nucleic Acids Res.

[46] Bolger AM, Lohse M, Usadel B (2014). Trimmomatic: a flexible trimmer for Illumina sequence data. Bioinformatics..

[47] Muturi EJ, Donthu RK, Fields CJ, Moise IK, Kim CH (2017). Effect of pesticides on microbial communities in container aquatic habitats. Sci Rep..

[48] Cole JR, Wang Q, Fish JA, Chai B, McGarrell DM, Sun Y (2014). Ribosomal Database Project: data and tools for high throughput rRNA analysis. Nucleic Acids Res..

[49] Hammer O, Harper DAT, Ryan PD (2001). PAST: Paleontological statistics software package for education and data analysis. Paleontol Electronica..

[50] Bokulich NA, Subramanian S, Faith JJ, Gevers D, Gordon JI, Knight R (2013). Quality-filtering vastly improves diversity estimates from Illumina amplicon sequencing. Nat Methods..

[51] McMurdie PJ, Holmes S (2013). phyloseq: an R package for reproducible interactive analysis and graphics of microbiome census data. PLoS One.

[52] Caporaso JG, Kuczynski J, Stombaugh J, Bittinger K, Bushman FD, Costello EK (2010). QIIME allows analysis of high-throughput community sequencing data. Nat Methods..

[53] Muturi EJ, Ramirez JL, Rooney AP, Kim CH (2018). Comparative analysis of gut gicrobiota of *Culex restuans* (Diptera: Culicidae) females from different parents. J Med Entomol..

[54] Zouache K, Raharimalala FN, Raquin V, Tran-Van V, Raveloson LH, Ravelonandro P, Mavingui P (2011). Bacterial diversity of field-caught mosquitoes, *Aedes albopictus* and *Aedes aegypti*, from different geographic regions of Madagascar. FEMS Microbiol Ecol..

[55] Ngo CT, Romano-Bertrand S, Manguin S, Manguin S, Jumas-Bilak E (2016). Diversity of the bacterial microbiota of *Anopheles* mosquitoes from Binh Phuoc Province, Vietnam. Front Microbiol..

[56] Akorli J, Gendrin M, Pels NA, Yeboah-Manu D, Christophides GK, Wilson MD (2016). Seasonality and locality affect the diversity of *Anopheles gambiae* and *Anopheles coluzzii* midgut microbiota from Ghana. PLoS One..

[57] Minard G, Mavingui P, Moro CV (2013). Diversity and function of bacterial microbiota in the mosquito holobiont. Parasit Vectors..

[58] Straif SC, Mbogo CN, Toure AM, Walker ED, Kaufman M, Toure YT, Beier JC (1998). Midgut bacteria in *Anopheles gambiae* and *An. funestus* (Diptera: Culicidae) from Kenya and Mali. J Med Entomol..

[59] Pumpuni CB, Demaio J, Kent M, Davis JR, Beier JC (1996). Bacterial population dynamics in three anopheline species: the impact on *Plasmodium* sporogonic development. Am J Trop Med Hyg..

[60] Fridman S, Izhaki I, Gerchman Y, Halpern M (2012). Bacterial communities in floral nectar. Environ Microbiol Rep..

[61] Ritpitakphong U, Falquet L, Vimoltust A, Berger A, Metraux JP, L’Haridon F. The microbiome of the leaf surface of *Arabidopsis* protects against a fungal pathogen. New Phytologist. 2016;210:1033–43. 10.1111/nph.13808 26725246

[62] Verhulst NO, Andriessen R, Groenhagen U, Bukovinszkine Kiss G, Schulz S, Takken W (2010). Differential attraction of malaria mosquitoes to volatile blends produced by human skin bacteria. PLoS One..

[63] Verhulst NO, Qiu YT, Beijleveld H, Maliepaard C, Knights D, Schulz S (2011). Composition of human skin microbiota affects attractiveness to malaria mosquitoes. PLoS One..

[64] Shi Y, Lou K, Li C. Growth and photosynthetic efficiency promotion of sugar beet (*Beta vulgaris *L.) by endophytic bacteria. Photosynth Res. 2010;105:5–13. 10.1007/s11120-010-9547-7 20405213

[65] Jung JY, Park MS, Kim SE, Park BH, Son JY, Kim EY (2010). Risk factors for multi-drug resistant *Acinetobacter baumannii* bacteremia in patients with colonization in the intensive care unit. BMC Infect Dis..

[66] Chavshin AR, Oshaghi MA, Vatandoost H, Pourmand MR, Raeisi A, Enayati AA (2012). Identification of bacterial microflora in the midgut of the larvae and adult of wild caught *Anopheles stephensi*: a step toward finding suitable paratransgenesis candidates. Acta Trop..

[67] Chavshin AR, Oshaghi MA, Vatandoost H, Yakhchali B, Zarenejad F, Terenius O (2015). Malpighian tubules are important determinants of *Pseudomona*s transstadial transmission and longtime persistence in *Anopheles stephensi*. Parasit Vectors..

[68] Chandler JA, Lang JM, Bhatnagar S, Eisen JA, Kopp A (2011). Bacterial communities of diverse *Drosophila* species: ecological context of a host-microbe model system. PLoS Genet..

[69] Cheli GH, Corley JC (2010). Efficient sampling of ground-dwelling arthropods using pitfall traps in arid steppes. Neotrop Entomol..

[70] Vink CJ, Thomas SM, Paquin P, Hayashi CY, Hedin M (2005). The effects of preservatives and temperatures on arachnid DNA. Invertebr Syst..

[71] Fouda MA, Hassan MI, Al-Daly AG, Hammad KM (2001). Effect of midgut bacteria of *Culex pipiens* L. on digestion and reproduction. J Egypt Soc Parasitol..

[72] Konig H (2006). *Bacillus* species in the intestine of termites and other soil invertebrates. J Appl Microbiol..

[73] Peck GW, Walton WE (2006). Effect of bacterial quality and density on growth and whole body stoichiometry of *Culex quinquefasciatus* and *Culex tarsalis* (Diptera: Culicidae). J Med Entomol..

[74] Minard G, Tran FH, Raharimalala FN, Hellard E, Ravelonandro P, Mavingui P, Moro CV (2013). Prevalence, genomic and metabolic profiles of *Acinetobacter* and *Asaia* associated with field-caught *Aedes albopictus* from Madagascar. FEMS Microbiol Ecol..

[75] Cirimotich CM, Dong Y, Clayton AM, Sandiford SL, Souza-Neto JA, Mulenga M, Dimopoulos G (2011). Natural microbe-mediated refractoriness to *Plasmodium infection* in *Anopheles gambiae*. Science..

[76] Osei-Poku J, Han C, Mbogo CM, Jiggins FM (2012). Identification of *Wolbachia* strains in mosquito disease vectors. PLoS One..

[77] Blagrove MSC, Arias-Goeta C, Failloux AB, Sinkins SP (2012). *Wolbachia* strain wMel induces cytoplasmic incompatibility and blocks dengue transmission in *Aedes albopictus*. Proc Natl Acad Sci USA..

[78] Rasgon JL, Scott TW (2003). *Wolbachia* and cytoplasmic incompatibility in the california *Culex pipiens* mosquito species complex: parameter estimates and infection dynamics in natural populations. Genetics..

[79] Stouthamer R, Breeuwer JAJ, Hurst GDD (1999). *Wolbachia pipientis*: Microbial manipulator of arthropod reproduction. Annu Rev Microbiol..

[80] Caragata EP, Real KM, Zalucki MP, McGraw EA (2011). *Wolbachia* infection increases recapture rate of field-released *Drosophila melanogaster*. Symbiosis..

[81] Peng Y, Nielsen JE, Cunningham JP, McGraw EA (2008). *Wolbachia* infection alters olfactory-cued locomotion in *Drosophila* spp. Appl Environ Microbiol..

