# Supplementary material for: Mosquito microbiota cluster by host sampling location
Source: Parasit Vectors. 2018 Aug 14;11:468. doi: 10.1186/s13071-018-3036-9 (PMC6092830; doi:10.1186/s13071-018-3036-9)
Supplement: Supplementary file 2 — Table S2. Number of OTUs from each phylum that were identified from nine mosquito species from six USA states. (DOCX 13 kb) [file 13071_2018_3036_MOESM2_ESM.docx]

**Additional file 2: Table S2.** Number of OTUs from each phylum that were identified from nine mosquito species from six USA states.

| Phylum/subphyla | Relative abundance (%) | # OTUs |
| --- | --- | --- |
| Acidobacteria | 0.02 | 1 |
| Actinobacteria | 10.22 | 42 |
| Bacteria | 0.64 | 11 |
| Bacteroidetes | 2.78 | 22 |
| Chlamydiae | 0.07 | 1 |
| Chloroflexi | 0.06 | 1 |
| Cyanobacteria | 0.39 | 7 |
| Deinococcus-Thermus | 0.51 | 5 |
| Elusimicrobia | 0.01 | 1 |
| Firmicutes | 14.14 | 48 |
| Fusobacteria | 0.03 | 1 |
| Planctomycetes | 0.03 | 3 |
| Alphaproteobacteria | 28.22 | 56 |
| Betaproteobacteria | 18.88 | 30 |
| Deltaproteobacteria | 0.87 | 2 |
| Epsilonproteobacteria | 0.50 | 2 |
| Gammaproteobacteria | 22.02 | 45 |
| Unclassified Proteobacteria | 0.19 | 3 |
| Tenericutes | 0.33 | 2 |
| Verrucomicrobia | 0.10 | 1 |
