# Supplementary material for: Mosquito microbiota cluster by host sampling location
Source: Parasit Vectors. 2018 Aug 14;11:468. doi: 10.1186/s13071-018-3036-9 (PMC6092830; doi:10.1186/s13071-018-3036-9)
Supplement: Supplementary file 3 — Table S3. Pairwise ANOSIM comparisons of sample groups. Only 12 pairwise comparisons (in bold) remained significant after adjusting for multiple comparisons. Abbreviations: PIP, Culex pipiens; QUA, Anopheles quadrimaculatus; VEX, Aedes vexans; MEL, Culiseta melanura; CON, Psorophora confinnis; MI, Michigan; IA, Iowa; MO, Missouri; MN, Minnesota; WI, Wisconsin; LA, Louisiana. Values on the upper half are global R-values while those on the lower half are the associated P-values. (DOCX 15 kb) [file 13071_2018_3036_MOESM3_ESM.docx]

**Additional file 3: Table S3.** Pairwise ANOSIM comparisons of sample groups. Only 12 pairwise comparisons (in bold) remained significant after adjusting for multiple comparisons. *Abbreviations*: PIP, *Culex pipiens*; QUA, *Anopheles quadrimaculatus*; VEX, *Aedes vexans*; MEL, *Culiseta melanura*; CON, *Psorophora confinnis*; MI, Michigan; IA, Iowa; MO, Missouri; MN, Minnesota; WI, Wisconsin; LA, Louisiana. Values on the upper half are global *R*-values while those on the lower half are the associated *P-*values.

|  | VEX_MI | VEX_IA | QUA_LA | MEL_WI | PIP_MO | PIP_MI | PIP_IA | PIP_MN | QUI_LA | TAR_IA | CON_LA |
| --- | --- | --- | --- | --- | --- | --- | --- | --- | --- | --- | --- |
| VEX_MI |  | 0.660 | 0.867 | 0.821 | 0.735 | -0.218 | 0.813 | 0.594 | 0.965 | 0.822 | 0.855 |
| VEX_IA | **0.0001** |  | 0.232 | 0.342 | 0.285 | -0.025 | -0.180 | 0.269 | 0.569 | 0.004 | 0.181 |
| QUA_LA | **0.0001** | 0.0596 |  | 0.251 | 0.406 | -0.006 | 0.123 | 0.265 | 0.625 | -0.049 | 0.117 |
| MEL_WI | **0.0001** | **0.0001** | 0.0065 |  | 0.366 | -0.100 | 0.036 | 0.285 | 0.475 | -0.130 | 0.108 |
| PIP_MO | **0.0001** | **0.0001** | 0.0062 | **0.0005** |  | 0.275 | 0.220 | 0.208 | 0.398 | 0.275 | 0.266 |
| PIP_MI | 0.9703 | 0.5194 | 0.4863 | 0.6988 | 0.1208 |  | 0.405 | 0.060 | 1.000 | 0.111 | 0.222 |
| PIP_IA | **0.0001** | 0.8495 | 0.1595 | 0.3977 | 0.1078 | 0.054 |  | 0.115 | 0.527 | 0.097 | 0.059 |
| PIP_MN | **0.0001** | **0.0001** | 0.0541 | 0.0084 | **0.0006** | 0.3738 | 0.238 |  | 0.452 | 0.056 | 0.223 |
| QUI_LA | 0.0019 | 0.0255 | 0.0395 | 0.0191 | 0.0799 | 0.1026 | 0.0925 | 0.0823 |  | 1.000 | 0.208 |
| TAR_IA | 0.0023 | 0.482 | 0.5731 | 0.725 | 0.135 | 0.2951 | 0.3374 | 0.3805 | 0.0995 |  | 0.124 |
| CON_LA | **0.0001** | 0.1177 | 0.1066 | 0.1437 | 0.0488 | 0.1219 | 0.1807 | 0.0904 | 0.319 | 0.2595 |  |
